# Disruption of Fractalkine Signaling Leads to Microglial Activation and Neuronal Damage in the Diabetic Retina

**DOI:** 10.1177/1759091415608204

**Published:** 2015-10-26

**Authors:** Sandra M. Cardona, Andrew S. Mendiola, Ya-Chin Yang, Sarina L. Adkins, Vanessa Torres, Astrid E. Cardona

**Affiliations:** 1 Department of Biology, South Texas Center for Emerging Infectious Diseases, The University of Texas at San Antonio, TX, USA

**Keywords:** CX3CR1, fractalkine, inflammation, microglia, neurodegeneration, retinal ganglion cell

## Abstract

Fractalkine (CX3CL1 or FKN) is a membrane-bound chemokine expressed on neuronal membranes and is proteolytically cleaved to shed a soluble chemoattractant domain. FKN signals via its unique receptor CX3CR1 expressed on microglia and other peripheral leukocytes. The aim of this study is to determine the role of CX3CR1 in inflammatory-mediated damage to retinal neurons using a model of diabetic retinopathy. For this, we compared neuronal, microglial, and astroglial densities and inflammatory response in nondiabetic and diabetic (Ins2^Akita^) CX3CR1-wild-type and CX3CR1-deficient mice at 10 and 20 weeks of age. Our results show that Ins2^Akita^ CX3CR1-knockout mice exhibited (a) decreased neuronal cell counts in the retinal ganglion cell layer, (b) increased microglial cell numbers, and (c) decreased astrocyte responses comparable with Ins2^Akita^ CX3CR1-Wild-type mice at 20 weeks of age. Analyses of the inflammatory response using PCR arrays showed several inflammatory genes differentially regulated in diabetic tissues. From those, the response in Ins2^Akita^ CX3CR1-deficient mice at 10 weeks of age revealed a significant upregulation of IL-1β at the transcript level that was confirmed by enzyme-linked immunosorbent assay in soluble retinal extracts. Overall, IL-1β, VEGF, and nitrite levels as a read out of nitric oxide production were abundant in Ins2^Akita^ CX3CR1-deficient retina. Notably, double immunofluorescence staining shows that astrocytes act as a source of IL-1β in the Ins2^Akita^ retina, and CX3CR1-deficient microglia potentiate the inflammatory response via IL-1β release. Collectively, these data demonstrate that dysregulated microglial responses in absence of CX3CR1 contribute to inflammatory-mediated damage of neurons in the diabetic retina.

## Introduction

Diabetic retinopathy (DR) is the most common microvascular complication of diabetes and is recognized as a leading cause of blindness worldwide (Gardner et al., 2002; Antonetti et al., 2012). Vascular abnormalities including increased vascular permeability leading to edema and neovascularization, breakdown of the blood-retinal barrier, and proliferation of endothelial cells are hallmarks of DR (Barber, 2003). Although abnormalities in the retinal vasculature are evident in DR, loss of cells in the neural retina supports the view of DR as neurodegenerative disease (Barber, 2003). Notably, the mechanisms underlying disease initiation and progression are not well understood. Moreover, the contribution of microglia to inflammatory-mediated damage to the retina is still unclear.

Normal vision requires coordinated cell–cell communication among resident retinal neurons, Müller cells, astrocytes, microglia, vasculature, photoreceptors, and pigmented epithelial cells. Microglia, the resident inflammatory cells of the central nervous system (CNS) and primary sensors of pathological damage act as a double-edged sword (Ransohoff and Cardona, 2010). Microglial functions can lead to neuroprotection or can cause neuronal injury (Benveniste, 1997; Aloisi, 2001; Ponomarev et al., 2005). Microglial activation has been observed in experimental models of early diabetic retinopathy (Gyurko et al., 2006; Liang et al., 2009), and *in vitro* studies suggest that glycated compounds that react with microglia contribute to cell activation and induce proinflammatory cytokine release (Tang and Kern, 2011).

The focus of this study is to understand the association of activated microglia to the cytokine milieu and neuronal damage during DR. For this, we utilize the fractalkine/CX3CR1, chemokine/chemokine receptor pair, as it provides a mechanism to modulate microglial activation. Fractalkine (CX3CL1 or FKN) exists on neuronal membranes and functions by signaling through its unique receptor CX3CR1 present on microglia (Jung et al., 2000; Cook et al., 2001). Although mainly produced in the CNS (Tarozzo et al., 2002, 2003), FKN is also predominant in peripheral endothelial cells (Rossi et al., 1998; Harrison et al., 2001). Several reports support the notion that FKN exerts an inhibitory signal on microglia (Cardona et al., 2006b; Raoul et al., 2008; Liu et al., 2010; Cho et al., 2011; Rogers et al., 2011). In humans, two single-nucleotide polymorphisms produce four allelic receptor variants (Faure et al., 2000; McDermott et al., 2000, 2001; Moatti et al., 2001; McDermott et al., 2003). Most individuals carry CX3CR1^V249/T280^, whereas CX3CR1^I249/M280^ is estimated in about 20% of the population. These changes decrease FKN affinity (McDermott et al., 2003), and several studies support a role for CX3CR1 in age-related macular degeneration (Craner et al., 2003; Tuo et al., 2004; Chan et al., 2005; Brion et al., 2011; Schaumberg et al., 2014), autoimmune uveitis (Dagkalis et al., 2009), and neurodegenerative models of Alzheimer’s disease and Parkinson’s disease. It is still yet to be determined how these changes affect microglial–neuronal communication and most importantly to what extent dysregulated microglial responses in absence of FKN signaling contribute to neuronal pathology in the diabetic retina.

To investigate the role of CX3CR1 in microglial function during DR, we crossed *Insulin2^Akita^* (*Ins2^Akita^*) mice, with CX3CR1-knockout (KO) mice, to study retinal pathology in nondiabetic and diabetic (*Ins2^Akita^*), wild type (WT or heterozygous [HET]), and CX3CR1-KO mice. The CX3CR1-deficient mouse strain was generated by disruption of the CX3CR1 gene via insertion of enhanced green fluorescent protein (GFP). These GFP knock-in reporter mice (Jung et al., 2000), *Cx3cr1^gfp/gfp^* or *Cx3cr1^−^^/^^−^* (CX3CR1-KO), are valuable to visualize microglial responses within intact tissues due to the fact that all microglia are green fluorescent in KO and HET mice (Cardona et al., 2006b). The Ins2^Akita^ mice contain a mutation in the insulin-2 gene that replaces cysteine at position 96 with tyrosine, leading to improper folding of the insulin molecule, and resulting in apoptosis of pancreatic beta-cells. Ins2^Akita^ mice are recognized as a relevant model of DR; they develop high glucose levels early in life (typically by 4–5 weeks of age), exhibit increased vascular permeability, and vision deficits are observed at chronic stages of diabetes (>20 weeks of age; Barber et al., 2005; Akimov and Renteria, 2012).

Analyses of nondiabetic and Ins2^Akita^ mice reveal that FKN levels in the retina are significantly decreased in Ins2^Akita^-KO mice. Quantification of microglia and neurons in the retina showed an increased number of retinal microglia in both Ins2^Akita^-WT and Ins2-^Akita^-KO mice at 10 weeks of age. Interestingly, Ins2^Akita^-KO microglia exhibited an activated morphological phenotype at both 10 and 20 weeks age, which was accompanied by the enhanced loss of retinal ganglion cells (RGCs) and increased level of IL-1β when compared with the Ins2^Akita^-WT group. The decrease in neuronal cell densities within the GCL was confirmed by neuronal staining using NeuN and TUJ1 antibodies. These results support the notion that FKN/CX3CR1 signaling maintains a protective microglial phenotype in the retina and suggest that dysregulated microglial responses lead to sustained IL-1β cytokine release and potentially be a critical mediator of long-term damage to RGCs during diabetes.

## Methods

### Mice

C57BL/6, *Cx3cr1^gfp/gfp^ (Cx3cr1^−^^/^^−^)*, and Ins2^Akita^ mice were obtained from the Jackson Laboratory and bred at the Laboratory Animal Resources Center facility at the University of Texas at San Antonio. C57BL/6 and *Cx3cr1^−^^/^^−^* were crossed with Ins2^Akita^ mice to generate diabetic *Cx3cr1^gfp/gfp^ (Cx3cr1^−^^/^^−^)* and *Cx3cr1^+/gfp^ (Cx3cr1^+/^^−^).* For all experiments Ins2^Akita^ mice are referred to as diabetic mice. Ins2^Akita^ mice carrying a wild-type CX3CR1 genotype are referred to Ins2^Akita^-WT mice, and similarly Ins2^Akita^ mice lacking CX3CR1 *(Cx3cr1^−^^/^^−^)* are referred to as Ins2^Akita^-KO mice. Males (5–20 weeks old) were used for all experiments due to the fact that Ins2^Akita^ female counterparts revealed inconsistent glycemic measurement in peripheral blood irrespective of the genotype of the CX3CR1 loci. All the mice were maintained at the Laboratory Animal Resources Center at the University of Texas at San Antonio and experiments were performed in accordance with National Institutes of Health guidelines and approved by the University of Texas at San Antonio Institutional Animal Care and Use Committee.

Mice were genotyped by polymerase chain reaction (PCR) using DNA from ear-punching and gene-specific primers, CX3CR1-X, CX3CR1-Y, and CX3CR1-Z, as previously described (Cardona et al., 2008) and Ins2^Akita^ primers AK-F: TgC TgA TgC CCT ggC CTg CT and AK-R: Tgg TCC CAC ATA TgC ACA Tg. Reactions were prepared in a volume of 10 µl using GoTaq Hot Start Green Master Mix (Promega). To identify Ins2 point mutation, PCR product was digested with Fnu4HI (New England Biolabs) restriction enzyme for 2 hr. CX3CR1 fragments were resolved in 1% agarose gels to visualize a 1 Kb band for WT (primers X and Y) and 1.2 Kb band for KO (primers Y and Z) PCR products, whereas Ins2 fragments were resolved in 3% agarose (2.5% LE Quick Dissolve agarose—GeneMate and 0.5% GenePure LowMelt Agarose—BioExpress) for better separation of Ins2 transgene (280 bp) and WT band (140 bp).

### Glycemic Measurements

To confirm the diabetic phenotype of Ins2^Akita^ mice, glucose level was measured using the Precision Xtra Kit. For this, a blood glucose test strip was inserted into the calibrated Precision Xtra meter, followed by the application of one drop of blood obtained from the submandibular vein of mice ranging from 5 to 20 weeks of age. Glucose levels 80–250 mg/dL are considered nondiabetic and levels >250 mg/dL are considered hyperglycemic.

### Antibodies

For immunohistochemistry experiments, tissues were stained with primary antibodies directed against Ionized Calcium-Binding Adapter Molecule 1 (IBA-1; as a microglia specific marker, 1:4,000; rabbit, Wako), IL-1β (1:200; rabbit, Abcam), glial fibrillary acidic protein (GFAP as an astrocytic marker, 1:4,000; Clone 2.2B10; rat, Invitrogen), NeuN (1:4000; clone: A60; mouse, Millipore), and Neuronal Class III β-Tubulin (1:1,000; clone: TUJ1; mouse, Covance). Secondary antibodies were purchased from the Jackson Laboratory and were as follows: Cy3-goat anti-rabbit, Cy5-donkey anti-rat, biotinylated-goat anti-mouse, biotinylated-goat anti-rabbit, and biotinylated antibodies were detected by using Cy3- or Cy5-conjugated streptavidin.

### Fractalkine Enzyme-Linked Immunosorbent Assay

For detection of soluble FKN in retinal tissues, mice (5–20 weeks old) were anesthetized with 5% isoflurane in an induction chamber, and following perfusion with ice-cold Hanks salt solution (HBSS, Invitrogen), eyes were enucleated, and retinas were dissected free from sclera/retinal-pigmented epithelium and disrupted manually using dounce homogenizers. Protein extracts were obtained by homogenization of two retinas in 300 µL of lysis buffer containing 150 mM NaCl, 0.01 M Tris-Base, 1 mM EDTA pH = 8.0, and protease inhibitor cocktail (Roche). Lysates were centrifuged at 9,000 × g for 5 min at 4℃, aliquoted, and stored at −80℃. Total protein concentrations were determined using the Bio-Rad protein reagent assay (Bio-Rad). Soluble fractalkine (CX3CL1) levels were detected in retinal extracts by enzyme-linked immunosorbent assay (ELISA) following the murine Duoset instructions (R&D Systems). Each sample was run in duplicate. Results were normalized to total protein and reported as picogram (pg) amounts of chemokine per milligram (mg) of protein.

### Serum Collection

Under 5% isoflurane anesthesia, and prior perfusion, blood was collected by cardiac puncture using a 1 mL tuberculin syringe and gently transferred to a microcentrifuge tube. Blood (200–300 µl) was allowed to clot at 4℃ for 4 hr and centrifuged at 2,000 × g for 20 min at 4℃. Serum was removed and stored at −20℃ in the presence of protease inhibitor cocktail (Roche) as previously described (Cardona et al., 2008).

### Multiplex Cytokine Assay

Cytokine production in retinal protein extracts and serum was analyzed using the Bio-Plex protein array system (Luminex-based technology; Bio-rad Laboratories, Hercules, CA). Briefly, following buffer perfusion, retinas were dissected and soluble retinal protein extracts were generated by manual dissociation as described earlier. Retina and serum samples were assayed for the presence of IL-1β, IL-2, IL-6, IL-10, TNF-α, IFN-γ, CCL2, and vascular endothelial growth factor (VEGF). Results were normalized to total protein and expressed as picogram of cytokine per milligram of protein. IL-2, IL-6, IFN-γ, and TNF-α levels were below detection limit in retinal tissues, and results are only discussed for IL-1β and VEGF.

### Nitrite Assay

Nitric oxide levels released from retinal homogenates were determined by measuring the accumulation of its stable degradation product, nitrite (Griess Assay; Sigma). Retinas were dissected and disrupted manually using dounce homogenizers. Cell suspensions were made in lysis buffer (0.3 mL/retina) as described earlier. Total nitrite levels in retinal supernatants (*n* = 3 mice per group) were measured in two different dilutions and run in duplicates. Results are reported as µM per milligram of protein.

### Retinal Whole Mounts, Immunofluorescence, and Microscopy

Eyes were enucleated from mice perfused with HBSS, followed by 4% parafolmadehyde (PFA). Eyes were postfixed in 4% PFA for 4 hr at 4℃. For isolation of the retina, cornea and iris were gently cut off; the lens was removed leaving a cup-like structure containing the retina in the inside and the retinal pigment epithelium (RPE) and the sclera on the outside. Minimizing any contact with the fragile retina, the RPE and sclera were gently separated, exposing the retina and leaving in its cup-like conformation. Prior to immunofluorescence staining, four incisions were made using spring scissors to create a four-petal structure that facilitated its flattening once mounted on the glass slides after staining was completed.

For immunofluorescence analyses, retinal whole mounts were blocked and permeabilized at 4℃ for 3 hr in 10% normal goat serum (NGS; Jackson Laboratory), containing 1% BSA (Sigma) and 0.3% Triton-X 100 (TX-100; Sigma) followed by incubation with primary antibody overnight at 4℃ in same buffer. Tissues were washed with phosphate-buffered saline (PBS) supplemented with 0.1% TX-100 and incubated with host-specific secondary antibodies for 2 hr at room temperature and mounted on slides with Fluorsave (Calbiochem) or Prolong gold (Life Technologies) reagent. Sections were imaged using a Zeiss LSM 510 Meta confocal microscope, equipped with an Argon 488 nm laser to image GFP, a HeNe 543 nm laser to detect Cy3-conjugated markers, a HeNe 633 nm laser to detect Cy5-conjugated markers, and a diodode 405 nm laser to image nuclear Hoechst staining. Images were acquired with a sequential line scan. Z-stack projections of the ganglion cell layer (GCL) and nerve fiber layer (NFL) where microglia, astrocytes, RGCs, and blood vessels are primarily concentrated and were processed using Imaris Software v7.2 (Bitplane). Imaris Coloc tool was used to ascertain the colocalization between two fluorochromes in an image. Briefly, the two channels of interest were selected, and an automatic threshold using the One Time Point setting (to calculate the intensity of the fluorochromes histogram for the entire 3D composition) was performed on both channels. Imaris Coloc volume statistics were used and data were graphed as the number of total colocalized voxels between two channels. For microglial cell counts, microglia were manually counted in six 20× images per retina and normalized to X, Y, and Z (i.e., 412 µm × 412 µm × 25 µm) coordinates of respective confocal z-stack and data expressed as microglia per mm^3^. Transformation index (TI) was used to characterize changes in microglial morphology as previously described (Fujita et al., 1996). Briefly, utilizing Image J analysis (NIH), TI was calculated by using the equation: [perimeter^2^/4π × area^2^]. TI was quantified in five microglia per image from four different mice and expressed as values ranging from 1 to 50 (a TI value of 1 represents a circular object). It is well recognized that microglial morphology is indicative of cellular function and utilizing TI, we observed amoeboid cells with fewer cellular processes had a TI value closer to 1 and higher TI values are indicative of ramified non-activated surveillant microglia.

### Cryosections and Tissue Staining

Mice were perfused with 1× HBSS, followed by 4% PFA. To facilitate removal of eyes with the optic nerves, the bones surrounding the eyes were cut prior enucleation as described earlier. Eyes were fixed in 4%PFA at 4℃ for 30 min and then passed through sequential solutions of 5%, 10%, 12.5%, and 15% sucrose for 30 min, followed by overnight incubation in 20% sucrose at 4℃. Tissues were then embedded in O.C.T. compound (Tissue-Tek) and stored at −80℃. Serial horizontal cryosections 10 µm in thickness (Shandon Cryostat, SME) were placed on SuperFrost Plus slides (Fisherbrand). Slides were wrapped in aluminum foil and stored at −80℃ or processed immediately for immunohistochemistry. Frozen sections were thawed for 60 min at room temperature and then hydrated in 0.1% BSA in PBS for 6 min. Cryosections were blocked for nonspecific Ig binding by incubation for 30 min in 10% NGS, at room temperature followed by incubation with primary antibody in 3% goat serum, overnight at 4℃. Slides were washed in 1× PBS with 1% BSA and incubated with host-specific secondary antibodies for 2 hr at room temperature in 3% BSA, followed by Streptavidin-Cy3 and Hoechst stain. Sections were mounted using Fluorsave (Calbiochem) reagent. Neuronal staining in cryosections was quantified in 15 images per mouse acquired with a Zeiss LSM 510 Meta confocal microscope, as described earlier.

### Isolation of Mononuclear Cells and Flow Cytometry

Brain mononuclear cells were isolated from diabetic-WT, diabetic-KO, and nondiabetic controls by homogenization of CNS tissue as previously described (Pino and Cardona, 2011). Retinal cell suspensions were generated from four to six retinas per sample following the Neural Dissociation Kit (P) protocol (Miltenyi-Biotec). Brain and retinal cellular suspensions were incubated with Fc block solution (anti-mouse CD16/CD32, clone 2.4G2; BD Pharmingen) followed by incubation for 30 min with a mix of fluorochrome-conjugated anti-mouse antibodies as follows. Antibody cocktail for brain mononuclear cells: CD45 APC-Cy7 (1:400; Clone 30-F11; BioLegend), CD11b PE (1:100; Clone M1/70; BD Pharmingen), CD11c PE-Cy7 (1:50; Clone N418; eBioscience), I-A/I-E PerCP (1:100; Clone M5/114.15.2; BioLegend), Ly6C Alexa-647 (1:50 Clone ER-MP20; Serotec), and Ly6G Pacific Blue (1:400; Clone 1A8; BioLegend). Antibody cocktail for retinal mononuclear cells: CD45 APC-Cy7 (Clone 30-F11; BioLegend), CD11b PE (Clone M1/70; BD Pharmingen), CD11c PE-Cy7 (Clone N418; eBioscience), I-A/I-E PerCP (Clone M5/114.15.2; BioLegend), CD206 MMR APC (1:50; Clone C068C2; BioLegend), and CD54 Pacific Blue (1:500; Clone YN1/1.7.4; BioLegend). Cells were washed with cell staining buffer (BioLegend), fixed in 2% PFA, and stored at 4℃ until flow cytometry reading. Samples were run at the flow cytometry core facility at the University of Texas at San Antonio using a four-laser LSR-II cytometer (BD Bioscience) and the FACS Diva software. Analysis was performed using FlowJo software v9.2.

### RNA Isolation, cDNA Preparation, and PCR Array

Mice were perfused with HBSS and total RNA was prepared from dissected retinal tissues using Trizol™ reagent (Invitrogen); samples were then extracted with chloroform and RNA precipitated with isopropanol as previously described (Cardona et al., 2006a). Total RNA was further purified using RNeasy Mini™ Kit (Qiagen) and on column DNase treatment. RNA quality was evaluated under agarose gel electrophoresis and RNA concentration was measured using a Nanodrop. After RNA was extracted and purified from the mouse retina, cDNA was generated from 2 µg of total RNA using the RT^2^ First Strand Kit (SABioscience) according to manufacturer’s instructions and diluted in RNase-free water. Then, the RT^2^ Profiler PCR Array (SABioscience) was performed by real-time PCR using an ABI 7900HT Sequence Detection System to check for expression of inflammatory-related genes (Supplementary Tables 1–3). The real-time PCR detection was carried out as following: The experimental cocktail was prepared by adding 550 µl of the 2× SABiosciences RT^2^ qPCR master mix and 448 µL ddH_2_O to 102 µL of the diluted cDNA mixture which was prepared from RT^2^ First Strand Kit (SABioscience). Then, 10 µL of this cocktail was added to each well of the 384-well PCR array. The array was then cycled on a real-time thermal cycler through the following program: one cycle for 10 min at 95℃ followed by 15 sec at 95℃ and 1 min at 60℃ for 40 cycles. SYBR green fluorescence was detected from each well during the annealing step of each cycle. Analyses of the raw data were done through the SABiosciences web-based PCR array data analysis software. The 384-well format of the PCR arrays includes four replicates of the 84 selected genes, 5 housekeeping genes, genomic DNA control, reverse transcription control, and positive PCR control. Plates were processed in an Applied Biosystems 7900 Fast Real-Time PCR System, using automated baseline and threshold cycle was set to 0.58. All quantifications (threshold cycle [*C_T_*] values) were normalized to that of glyceraldehyde-3-phosphate dehydrogenase (GAPDH) and analyzed to determine the relative level of gene expression. Comparisons between groups were made using Student’s *t* tests with the level of significance set at *p* < .05. This analysis was performed using the SABiosciences web-based PCR array analysis data analysis tool (http://pcrdataanalysis.sabiosciences.com/pcr/arrayanalysis.php). The relative gene expression of the genes was calculated as ΔCt sample = (Ct sample GENE) − (Ct sample HKG). Normalized data were then compared between groups (Supplementary Table 4). Each gene fold-changes were calculated as difference in gene expression between each genotype. A positive value indicated gene up-regulation while a negative value indicated gene down regulation. Fold changes are presented only for genes that generated a *p* value < .05 when comparing among groups.

### Statistical Analysis

All measurements were plotted as mean ± *SEM* and bar graphs were overlaid with scatter plots to show sample distribution. Statistical tests were performed in GraphPad Prism v5.0. When appropriate, the unpaired Student’s *t* test (two-tailed) was used to analyze data between two groups, or for multiple comparisons, a one-way ANOVA with the Tukey’s multiple comparison test was performed. Significant differences were defined as *p* < .05 unless otherwise stated. The *p* values for the PCR array data were calculated based on a Student *t* test of the replicate 2(−Delta Ct) values for each gene in the control and experimental groups.

## Results

### Diabetic Model to Study the Role of CX3CR1 on Neuronal Pathology

**Figure 1.**
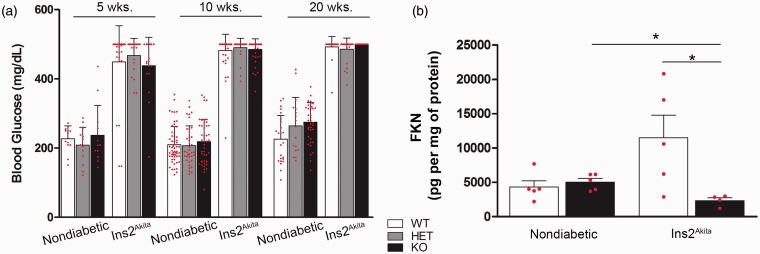
*Cx3cr1^gfp/gfp^* mice represent a model to investigate CX3CR1 signaling in *Ins2^Akita^* diabetic mice. (a) Blood glucose levels were compared in *Ins2^Akita^* mice on a CX3CR1 wild type (WT), heterozygous (HET), or knock-out (KO) background. (b) FKN levels were measured in retinal protein extracts at 10 weeks of age by ELISA in nondiabetic and Ins2^Akita^-WT and KO mice. *p < 0.05. Each point represent a data value from an individual mouse, *n* = 4–5 mice per group.

We developed a diabetic model in which the CX3CR1 mutation was introduced into Ins2^Akita^ transgenic mice. Both, Ins2^Akita^-WT and Ins2^Akita^-KO mice displayed high glucose levels (>250 mg/dL) by 5 weeks of age (Figure 1(a)). The hyperglycemic phenotype was sustained by 20 weeks of age (Figure 1(a)) and accompanied by excessive urination, a typical symptom of diabetes. This result is in agreement with previous studies showing no differences in glycated hemoglobin between Ins2^Akita^-WT and -KO mice (Kezic et al., 2013). Moreover, our previous studies have indicated that FKN is expressed constitutively in brain and spinal cord tissues in neurons as a transmembrane protein (Huang et al., 2006; Cardona et al., 2008). Constitutive cleavage of the soluble chemokine domain of FKN leads to basal levels of expression and exert its inhibitory actions by signaling through CX3CR1 on microglial cells (Re and Przedborski, 2006). We sought then to investigate FKN expression in the retina and determine differences between Ins2^Akita^ and nondiabetic mice. We utilized ELISA to detect the soluble FKN levels in retinal aqueous protein homogenates. Since FKN is mainly produced by neurons, the observation of decreased soluble FKN (Figure 1(b)) in retinal tissues of Ins2^Akita^-KO mice (4,337 ± 908 pg/mg of protein in nondiabetic WT mice vs. 11,503 ± 3,321 in Ins2^Akita^-WT; and 5,058 ± 525 in nondiabetic-KO group vs. 2,373 ± 400 in Ins2^Akita^-KO, *p* = .0159 when comparing Ins2^Akita^-KO vs. nondiabetic KO, and *p* = .0317 between Ins2^Akita^-WT and Ins2^Akita^-KO) suggested a potential dysregulated response and prompt us to analyze in more detail microglia and neuronal distribution in our model and the contribution of CX3CR1 to the inflammatory reaction in the diabetic retina.

### Disruption of FKN Signaling Alters Microglial Numbers and Correlates With Decreased Neuronal Densities

**Figure 2.**
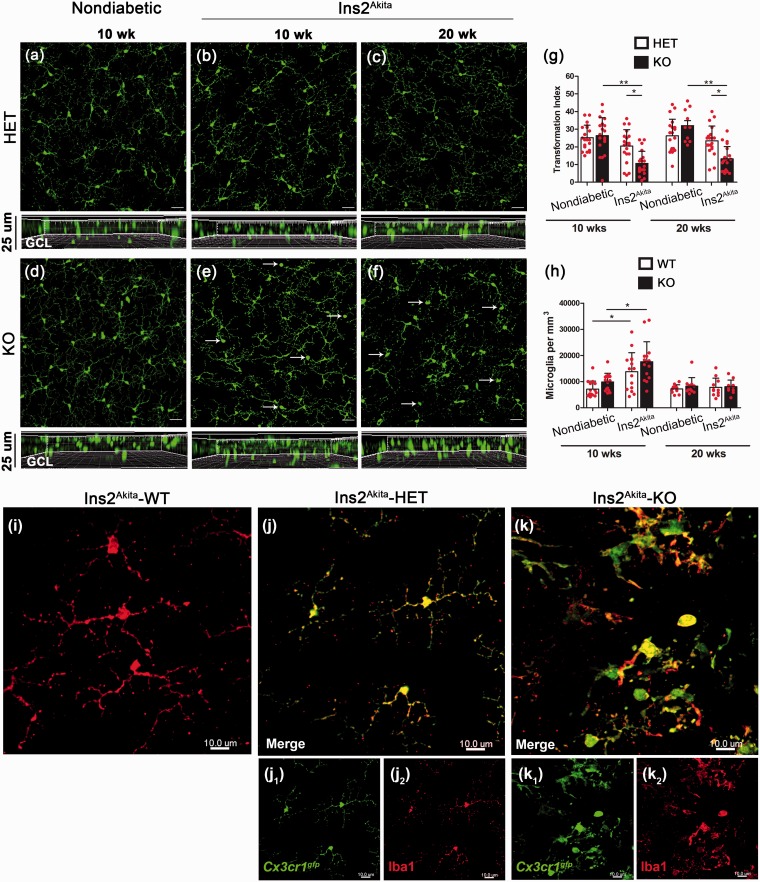
Increased numbers of retinal microglia in diabetic mice at 10 weeks of age and enhanced morphological activation in mice lacking CX3CR1. Microglia were imaged by confocal microscopy and analyzed in retinal whole mounts by virtue of GFP expression in CX3CR1-HET (a–c) and CX3CR1-KO mice (d–f, arrows point to amoeboid microglia). Nondiabetic tissues (a and d) were compared with retinas isolated from Ins2^Akita^ mice at 10 (b and e) and 20 weeks (c and f) of age for microglial activation based on morphology changes and quantification of the transformation index (g). Panels below images (a–f) show z-stack composition in the GCL. Microglial cells were counted in retinal tissues and compared among nondiabetic and diabetic WT and KO mice (h). *n* = 5 mice per group. *p < 0.05, **p < 0.01. Microglia exhibit comparable morphology and distribution in both Ins2^Akita^–WT (i) and Ins2^Akita^-HET (j) mice based on IBA-1 staining (i,j2). Higher magnification of microglia in Ins2^Akita^–KO mice clearly depicts the activated state based on microglia morphology (k) in comparison to Ins2^Akita^-WT (i) and Ins2^Akita^-HET (j). Scale bar (a–f), 30 µm.

Evidence from experimental models and tissues from DR patients indicate that microglia undergo changes in morphology, location, and numbers in the diabetic retina. However, it is unclear how activation of microglia evaluated by changes in cellular morphology, proliferation or mobilization correlate with pathological changes in retinal neurons. To address this, we determined microglial numbers in whole retina mounted tissues using confocal imaging at 10 and 20 weeks of age. In nondiabetic CX3CR1-HET and CX3CR1-KO mice, microglia were the only cells expressing the GFP-reporter and microglia distribution and numbers in WT mice based on IBA-1 staining was comparable with CX3CR1-HET mice. Microglia from nondiabetic CX3CR1-HET mice (Figure 2(a)) showed a uniform distribution throughout the retina and hyperglycemia in these mice did not seem to dramatically alter microglial morphology (Figure 2(b) and (c)), a parameter associated with cellular activation. More specifically, CX3CR1-HET microglia in nondiabetic and Ins2^Akita^ mice (Figure 2(a)–(c)) displayed small cell bodies and long/thin cellular processes, confirmed both by GFP and IBA-1 expression (2j1, j2). Note the comparable morphology and distribution of HET and WT microglia (Figure 2(i) and (j)). A similar pattern was observed in microglia from nondiabetic CX3CR1-KO retinas (Figure 2(d)). However, microglia of Ins2^Akita^-KO mice showed a time-dependent progression toward an amoeboid phenotype, with more cells displaying thicker and shorter processes by 20 weeks of age (Figure 2(e) and (f)) compared with milder morphological changes in the Ins2^Akita^-HET group (Figure 2(b) and (c)). To quantify morphological changes in microglia, we used the transformation index (TI) as a measurement of cell shape taking into account measurements of cell perimeter and area. Cells with longer and thinner processes display higher cell perimeters that will correlate with higher TI values. In contrast, cells that are small and round will have lower TI values. Our results indicate that microglia in nondiabetic mice at 10 and 20 weeks of age exhibit comparable morphologies. However, Ins2^Akita^-CX3CR1-deficient mice have statistically significant lower TI values indicative of a more activated/amoeboid phenotype (Figure 2(g)). IBA-1 staining of Ins2^Akita^*-*KO microglia also reflects its activated morphology when colocalized to GFP (Figure 2(k)). Ins2^Akita^-WT and Ins2^Akita^-HET microglia exhibit a similar morphology and distribution supporting the rationale that WT and HET microglia function is readily comparable and can be attributed an undistinguishable phenotype in retinal tissues. Of interest, microglial cell numbers in the GCL did not show significant differences, except at 10 weeks of age (Figure 2(h)). Both Ins2^Akita^-WT and Ins2^Akita^-KO mice at 10 weeks of age showed an increase in microglial densities with a more pronounced increase in the Ins2^Akita^-KO group (Figures 2(h)).

**Figure 3.**
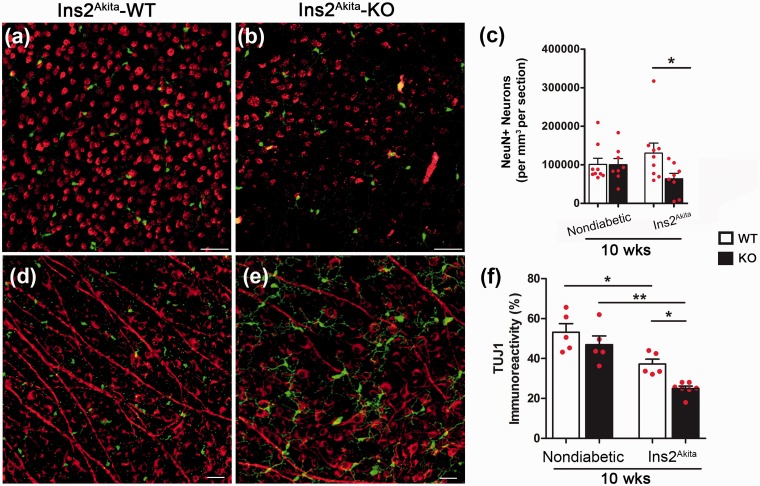
Decreased neuronal counts is associated with absence of CX3CR1 in diabetic retina. NeuN-positive neurons were quantified in retinal whole mounts (a–c; NeuN in red staining, and microglia in green). A pronounced reduction of neuronal cells is observed in Ins2^Akita^-KO mice in comparison to Ins2^Akita^-WT mice when total neurons are quantified in GCL (c). To confirm these finding, whole retinal tissues were stained against neuronal beta tubulin class 3 (TUJ1) antibodies (d–f; TUJ1: red staining and microglia in green) to compare staining of cell bodies and axons of RGCs within the GCL. *n* = 5 mice per group. *p < 0.05, **p < 0.01. In panels (c) and (f), white bars represent data from CX3CR1-WT and black bars from CX3CR1-KO mice. Scale bar (a) and (b), 50 µm; (d) and (e), 30 µm.

We next determined the association between the microglial phenotype and neuronal cell numbers. For this, we initially quantified the number of NeuN-positive cells within the RGC layer using cryosections (data not shown) obtained from nondiabetic and diabetic groups at 10 and 20 weeks of age. To sample enough retinal volume, positive cells were counted in 15 sections per mouse and the results showed a decreased in the number of NeuN-positive cells in Ins2^Akita^-WT and Ins2^Akita^-KO groups when compared with their nondiabetic controls at both 10 and 20 weeks of age. Given the limitation in imaging the entire retina in eyes that have been cryosectioned and in efforts to capture the entire layer of RGCs, we then pursued another approach in which we stained the whole retina and imaged six fields in the peripheral retina where most cell bodies are located. Since microglial numbers appeared significantly increased at 10 weeks of age, we then quantified the number of neurons at the same time point in the RGC layer using immunofluorescent staining in retinal whole mounts with antibodies against NeuN (Figure 3(a)–(c)) and TUJ1 (Figure 3(d)–(f)). Total number of NeuN-positive neurons per volume, which include the entire RGC, showed a significant decrease in Ins2^Akita^-KO mice when compared with Ins2^Akita^-WT (Figure 3(c); NeuN counts per volume in Ins2^Akita^-WT = 130,457 ± 25,840 and 63,724 ± 14,311 in Ins2^Akita^-KO, *p* = .036). Similarly, retinal whole mount staining against TUJ1 revealed a statistically significant decrease in the immunoreactive area between Ins2^Akita^-WT and Ins2^Akita^-KO groups (Figure 3(f); TUJ1 immunoreactive area in Ins2^Akita^-WT group = 37.20 ± 5.60 vs. 24.97 ± 3.40 in Ins2^Akita^-KO, *p* = .0014). TUJ1 staining not only shows an overall decreased in neuronal cell bodies but interestingly, the Ins2^Akita^-KO groups exhibit a decreased axonal staining. Overall, these results support the involvement of neuronal damage to the pathology of DR in experimental models lacking CX3CR1.

### Microglia Are the Predominant Resident Immune Cell Population in the Retina and Diabetic CX3CR1-Deficient Mice Exhibit a Predominant Proinflammatory Phenotype

**Figure 4.**
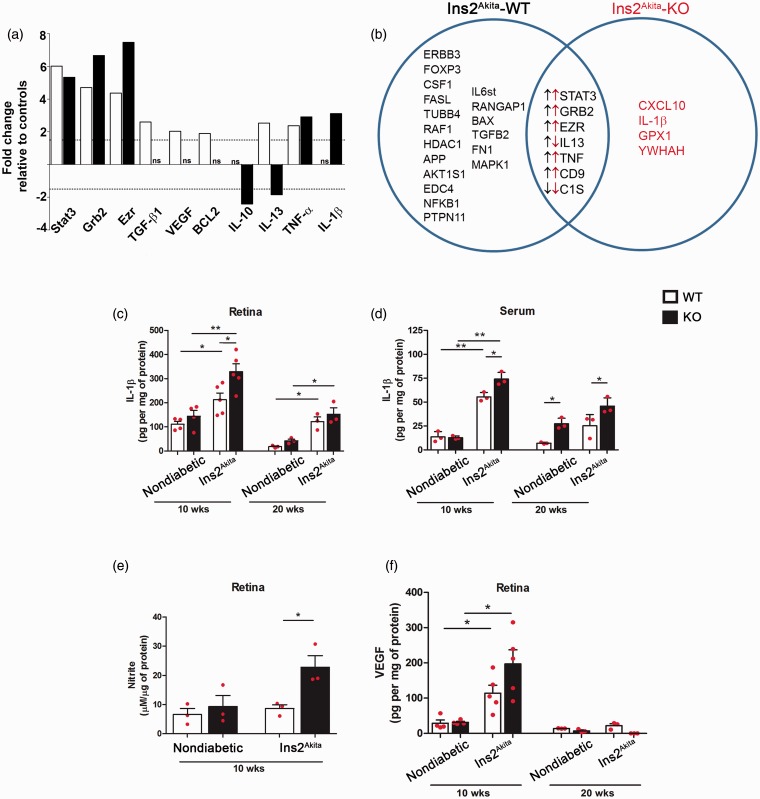
Predominant expression of IL-1β in diabetic CX3CR1-KO retina. (a) Gene expression of selected genes statistically significant when comparing the Ins2^Akita^-WT to their nondiabetic-WT controls (white bars) and Ins2^Akita^-KO compared with nondiabetic-KO controls (black bars) are shown. (b) List of all genes that were statistically significant and uniquely upregulated in the respective group when comparing Ins2^Akita^-WT versus Ins2^Akita^-KO are presented in the Venn diagram. List of common/shared genes relative to their nondiabetic controls are shown in the overlapped area (black and red arrows indicate upregulation or downregulation in Ins2^Akita^-WT and Ins2^Akita^-KO groups, respectively). IL-1β expression levels quantified by ELISA in retinal protein extracts (c) and serum (d) were compared at 10 and 20 weeks of age, and nitrite levels as a read out of nitric oxide levels were measured at 10 weeks of age (e) in nondiabetic controls and Ins2^Akita^ WT and KO groups. In addition, VEGF levels were measured in retinal protein extracts in Ins2^Akita^ and nondiabetic groups at 10 and 20 weeks of age (f). Data from mice on a CX3CR1-WT background are shown in the white bars, and black bars indicate the data from mice in a CX3CR1-KO background. *p < 0.05, ** *p* < 0.01. Each point represents a data value from an individual mouse.

To address the role of recruitment of blood leukocytes to the retinal pathology observed in hyperglycemic mice, we sought to compare myeloid cell populations by flow cytometry. Hematogenous CD45^Hi^ leukocytes and CD45^Lo^ microglia proportions were compared in retina (Supplementary Figure 1) and brain tissues of nondiabetic and Ins2^Akita^ mice (data not shown). Overall, no significant differences were observed in the relative frequencies of CD45^Hi^ and CD45^Lo^ populations among the groups. Retinal and brain microglia did not exhibit statistically significant changes in expression of MHC-II, CD11c, and CD54, and detailed analyses of other myeloid cell population did not reveal a significant contribution of hematogenous cells during this model (Data not shown). Therefore, we focused our investigation in identifying inflammatory-related genes involved in DR, by comparing gene expression in CX3CR1-WT, CX3CR1-KO, Ins2^Akita^-WT, and Ins2^Akita^-KO mice by quantitative real-time PCR. We used a highly sensitive PCR-based array system containing 84 key genes related to inflammatory diseases (Qiagen, 330231, Supplementary Tables 1–3) in nondiabetic and Ins2^Akita^ mice at 10 and 20 weeks of age. Normalized results were compared first among nondiabetic-WT and nondiabetic-KO groups, and no major changes in inflammatory response were detected (Supplementary Tables 4 and 5) indicating that in a nondiabetic state there are no significant differences between WT and KO retinal tissues at 10 and 20 weeks of age. Differentially expressed genes in Ins2^Akita^-WT and Ins2^Akita^-KO are summarized in Table 1. Interestingly, common transcripts upregulated in both groups included, signal transducer and activator of transcription (STAT3), growth factor receptor bound protein-2 (Grb2), and Ezrin where highly upregulated in both Ins2^Akita^-WT (Figure 4; white bars and Supplementary Table 6) and Ins2^Akita^-KO (Figure 4(a); black bars and Supplementary Table 7) groups. However, transcripts for the anti-inflammatory cytokines TGF-β1 and IL-13, TNF-α, and the anti-apoptotic protein Bcl-2 were upregulated in Ins2^Akita^-WT mice (Figure 4(a)) when compared with nondiabetic controls. In contrast, comparison of Ins2^Akita^-KO mice (to nondiabetic-KO mice) at 10 weeks of age revealed increased transcript levels for the proinflammatory cytokines TNF-α and IL-1β, whereas transcripts for the anti-inflammatory cytokines IL-10 and IL-13 were down regulated (Figure 4(a) and Supplementary Table 7). Although the response in Ins2^Akita^-KO mice at 10 weeks of age appears dominated by IL-1β response (Figure 4(b)), at 20 weeks of age (Supplementary Tables 8–10) the pattern of expression did not reveal a particular trend but rather a shared mix of pro- and anti-inflammatory transcripts in both diabetic groups.

**Table 1.** Differentially Expressed Genes in Ins2^Akita^-KO Versus Ins2^Akita^-WT Group at 10 Weeks of Age.

Gene	Description or general function	Fold change^a^	Summary
CXCL10	Chemokine (C-X-C motif) ligand 10, CXCR3 receptor, chemoattractant for monocytes and T cells	2.1973	↑ in Ins2^Akita^-KO
IL1b	Proinflammatory cytokine; associated with neurotoxicity	1.5005	
GPX1	Glutathione peroxidase 1; detoxification of hydrogen peroxide	1.2901	
YWHAH	Tyrosine 3-monooxygenase/tryptophan 5-monooxygenase activation protein, eta polypeptide; metabolisms, protein trafficking, signal transduction, apoptosis, and cell-cycle regulation	1.168	
MAPK1	Mitogen-activated protein kinase 1; signal transduction	−1.2372	↑ in ins2^Akita^-WT
FN1	Fibronectin 1; cell adhesion and migration, binds collagen, fibrin, heparin, and actin	−1.3588	
TGFB2	Transforming growth factor, beta 2; associated with neovascular glaucoma and macular holes	−1.3698	
BAX	Bcl2-associated X protein; proapoptotic	−1.3817	
RANGAP1	RAN GTPase activating protein 1; convers Ran to the putatively inactive GDP-bound state	−1.3905	
IL6ST	IL6 signal transducer, initiate signal transmission for IL6, LIF, CNTF, IL11, etc.	−1.3965	
PTPN11	Protein tyrosine phosphatase, nonreceptor type 11; perturbations implicated in type II diabetes, ablation results in progressive apoptosis of retinal cell types	−1.4018	
NFKB1	Nuclear factor of kappa light polypeptide gene enhancer in B-cells 1, p105; inflammatory signal transduction	−1.4435	
EDC4	Enhancer of mRNA decapping 4; mRNA degradation	−1.452	
AKT1S1	AKT1 substrate 1 (proline-rich); subunit of mTORC1 negatively regulates mTOR activity	−1.4668	
APP	Amyloid beta (A4) precursor protein; major component of amyloid plaques	−1.5215	
HDAC1	Histone deacetylase 1, epigenetic transcriptional regulation	−1.5378	
RAF1	V-raf-leukemia viral oncogene 1; signal transduction, proliferation, survival	−1.5552	
MAP2K1	Mitogen-activated protein kinase kinase 1	−1.6423	
TUBB4	Tubulin, beta 4; scaffold protein to determine cell shape and organelle/vesicle movement	−1.8292	
FASL	Fas ligand (TNF superfamily, member 6); protects against apoptosis	−1.9673	
CSF1	Colony stimulating factor 1 (macrophage); survival, proliferation and differentiation of myeloid cell precursors (microglia, monocytes, macrophages)	−2.0031	
FOXP3	Forkhead box P3; transcriptional regulator	−2.4351	
ERBB3	V-erb-b2 erythroblastic leukemia viral oncogene homolog 3 (avian); signal transduction	−2.4637	

a Positive value indicate up regulation in Ins2^Akita^-KO group and negative values in Ins2^Akita^-WT group. Summary of this table is represented as a Venn diagram in Figure 4(b).

To confirm the relevance of IL-1β, one of the cytokines uniquely upregulated in Ins2^Akita^-KO retinas, we performed IL-1β ELISA in retinal protein homogenates. At 10 weeks of age, there was a significant increase in the levels of IL-1β in Ins2^Akita^-KO mice when compared with their nondiabetic counterparts. Although IL-1β was not detected at statistically significant levels by qRT-PCR in Ins2^Akita^-WT mice, the ELISA results showed that hyperglycemia upregulates IL-1β in WT mice. Most notably, the levels of IL-1β are significantly higher in Ins2^Akita^-KO mice when compared with the Ins2^Akita^-WT group (Figure 4(c)). Serum levels of IL-1β were also abundant in Ins2^Akita^-WT and Ins2^Akita^-KO mice when compared with nondiabetic mice, but significantly higher in the Ins2^Akita^-KO group, and overall, retinal and circulating levels of IL-1β decreased by 20 weeks of age (Figure 4(d)). Since IL-1β has been reported to induce nitric oxide (Corbett et al., 1992; Lima-Junior et al., 2013) and both can contribute to inflammation and oxidative stress, we then sought to determine differences in levels of nitric oxide between the groups at 10 weeks of age. For this, we used the Griess reagent to detect nitrite, a final product of nitric oxide oxidation pathway and found that retinal tissues of Ins2^Akita^-KO mice exhibited higher nitrite levels (Figure 4(e)). In Ins2^Akita^-WT retinas, nitrite levels did not correlate to the IL-1β response. The complex biology of nitric oxide production prompts further investigation on how FKN/CX3CR1 signaling, IL-β, and enzymes involved in nitric oxide production converge during the pathology of DR.

Another molecule of particular interest in DR pathology is VEGF. In our study, transcript levels did not correlate with the protein levels. This observation highlights the importance of regulatory controls, and also prompts validation of expression. Based on levels detected by ELISA, hyperglycemia upregulates VEGF expression in both Ins2^Akita^-WT and Ins2^Akita^-KO retinas. Interestingly, VEGF levels were increased at 10 weeks of age in Ins2^Akita^-KO and Ins2^Akita^-WT retinal tissues (Figure 4(f)), highlighting the potential involvement of an increase vascular pathology earlier during disease that was ameliorated at the 20-week time point. These results suggest that IL-1β in combination with high nitric oxide levels may contribute to neuronal and axonal damage during DR.

### CX3CR1-Deficient Microglia Produce IL-1β and Contribute to the Inflammatory Reaction in the Retina

**Figure 5.**
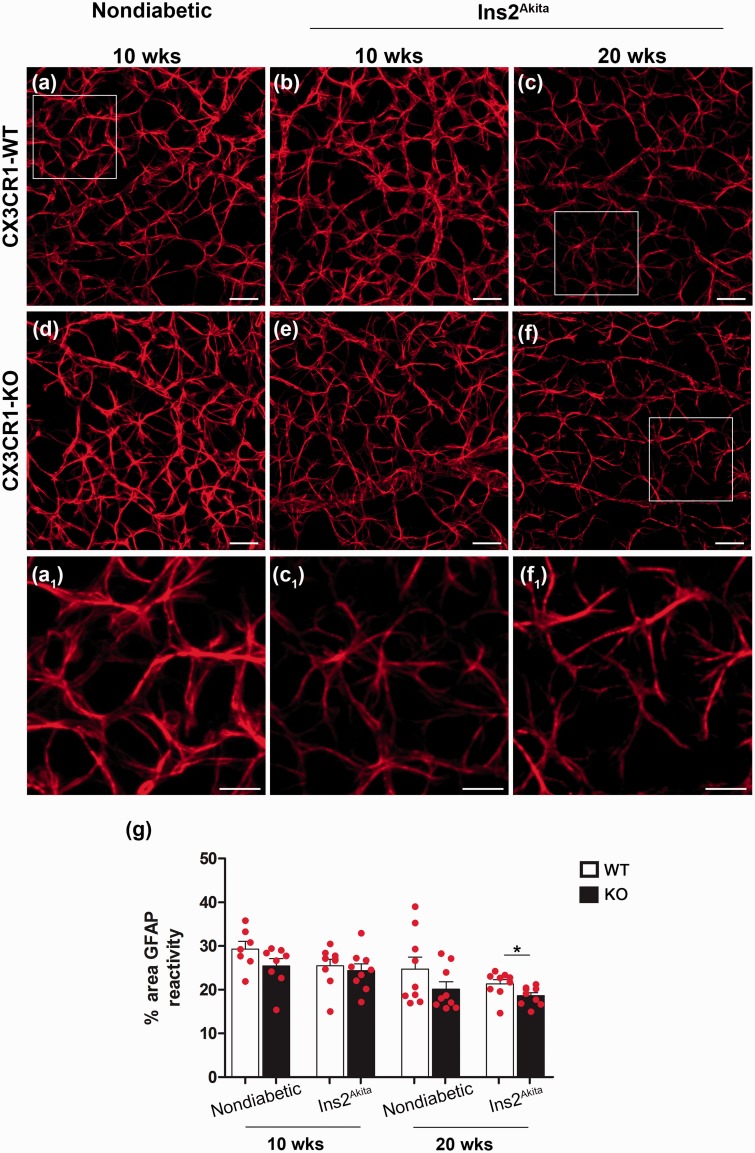
Astrocyte response in the retina. GFAP-positive cells (red fluorescence) were imaged in retinal sections within the GCL and NFL in CX3CR1-WT (a–c) and CX3CR1-KO mice (d–f). Nondiabetic control tissues (a and d) were compared with Ins2^Akita^ mice at 10 (b and e) and 20 weeks of age (c and f) and data presented as immunoreactive area occupied by GFAP staining (g). Higher magnification images show a reduction in the complexity of GFAP processes in Ins2^Akita^-KO (f1) mice at 20 weeks of age vs. nondiabetic WT (a1) and Ins2^Akita^-WT (c1) mice. *n* = 3 mice per group. *p < 0.05.

**Figure 6.**
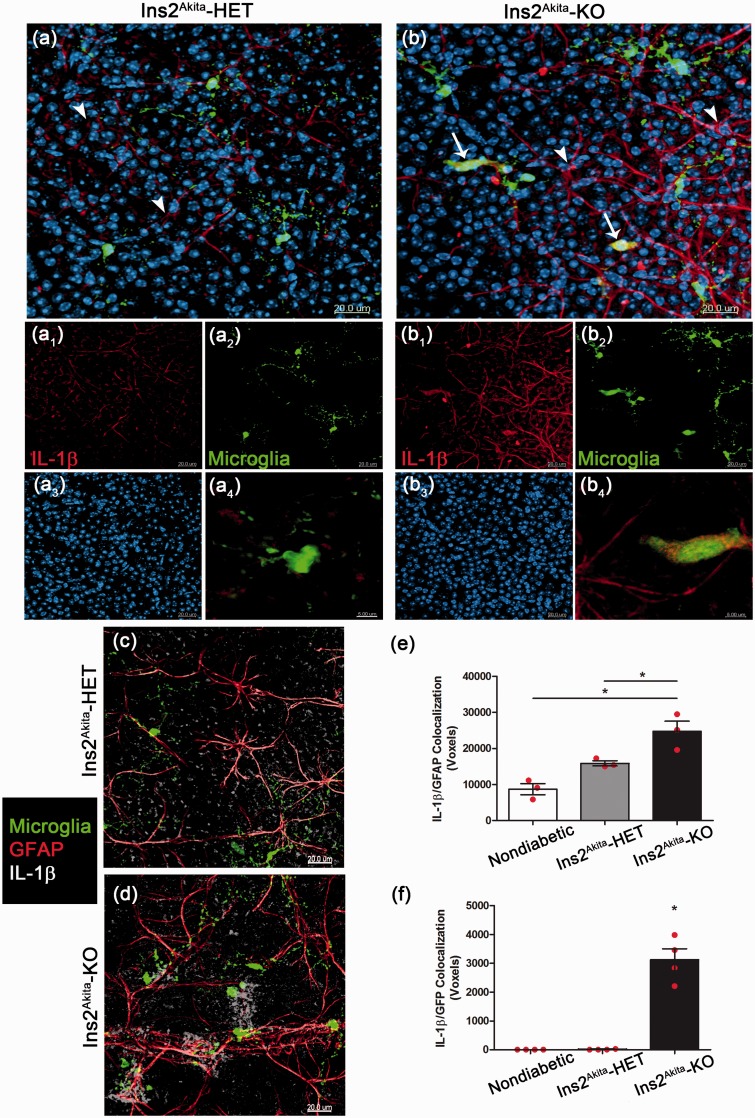
CX3CR1-deficient microglia produce IL-1β and contribute to the inflammatory reaction in the diabetic retina. Retinal whole mounts were stained and imaged for IL-1β (red) and microglia (green) in Ins2^Akita^-HET (a, arrowheads point to IL-1β staining) and Ins2^Akita^-KO tissues (b, arrows point to microglia positive for IL-1β staining). Images a and b show merged channels (a1 and b1 show IL-1β in red, a2 and b2 show microglia in green, and a3 and b3 nuclear staining). Higher magnification panels a4 and b4 show differential IL-1β expression in Ins2^Akita^-HET and Ins2^Akita^-KO microglia, respectively. Double immunofluorescence for IL-1β and the astroglial marker GFAP (c and d) in conjunction with the CX3CR1-GFP microglial reporter were used to compare IL-1β production in astrocytes (e) and microglia (f) in nondiabetic and Ins2^Akita^-HET and Ins2^Akita^-KO mice using Imaris software. The number of double positive voxels in the IL-1β/GFAP channels (e) and IL-1β/Microglia (f) channels are shown. *p < 0.05.

Given the strong correlation between microglia activation, a biased proinflammatory phenotype and decreased neuronal cell counts in CX3CR1-deficient diabetic mice, we next determined the role of CX3CR1-KO microglia as a key source of IL-1β. Retinal whole mounts from nondiabetic and Ins2^Akita^ mice at 10 weeks of age were stained with antibodies against IL-1β. Spleen tissues were used as positive controls in addition to appropriate negative controls without primary antibodies (Supplementary Figure 2). In agreement with the ELISA results, nondiabetic (WT and KO) tissue staining revealed low levels of IL-1β in the retina (Supplementary Figure 3), and immunoreactivity is detected mostly in cells that are GFAP positive. Overall comparison of astroglial responses based on GFAP staining within the NFL (which excludes the Müller cell bodies) did not reveal significant differences among nondiabetic and diabetic groups at 10 weeks of age. However, decreased GFAP immunoreactivity was observed at 20 weeks of age in Ins2^Akita^-KO mice when compared with Ins2^Akita^-WT mice (Figure 5(f), (f1), (c), and (g)). Particularly, astrocytes did not look as ramified as seen at 10 weeks of age (Figure 5(b) and (e)). However, when analyzing the IL-1β response in the diabetic retina, a significant upregulation was detected in cells with morphological features of astrocytes in Ins2^Akita^-KO mice (Figure 6(a)–(b)). Double immunofluorescence for the astroglial marker GFAP confirms that in Ins2^Akita^ mice, astrocytes within the NFL are a predominant source of IL-1β (Figure 6(c)–(e)). Interestingly, in Ins2^Akita^-KO mice microglia also contribute to IL-1β production (Figure 6(d)–(f)). Analysis of double fluorescence colocalization between GFAP and IL-1β staining and microglial GFP and IL-1β showed the unique contribution of CX3CR1-KO microglia to the inflammatory reaction in the retina, whereas microglia in diabetic-WT mice did not appear to be a major contributor to IL-1β release. These results suggest that dysregulation of microglial responses in the absence of CX3CR1 signaling contribute to neuronal pathology in the diabetic retina.

## Discussion

Although several treatments are available for DR, including surgical vitrectomy or intraocular injection of VEGF inhibitors and steroids, and photocoagulation, these alternatives are not successful for early stages of DR and do not completely eliminate the risk of vision loss (Cheung et al., 2010). To develop novel interventions for the management of DR, some important questions need to be addressed. One of them is the potential role of inflammation in neuronal degeneration in the diabetic retina. This study was conceived based on previous data showing increased microglia activation in mice lacking CX3CR1 and in which a predominance of proinflammatory mediators correlated with neurotoxicity in the brain in models of low-level endotoxemia, Parkinson’s disease, and Amyotrophic lateral sclerosis (Cardona et al., 2006b). For this, we used the Ins2^Akita^ mouse model that spontaneously develops hyperglycemia as early as 4 weeks of age and in which retinal cell apoptosis has been identified (Gastinger et al., 2008). To expand these studies, we generated Ins2^Akita^ mice in a CX3CR1-WT, HET, or KO background to address the potential role of microglia-mediated inflammation in neuronal damage in the retina. Our data extend the findings of neuronal reduction in the diabetic retina using the specific neuronal markers NeuN and TUJ1. The reduction in neuronal densities correlated with morphological microglial activation, and the present study provides novel insights into the inflammatory reaction in the context of diabetes using the Ins2^Akita^ model and dysregulated microglial responses in absence of CX3CR1 signaling.

In the search for differentially regulated inflammatory genes in the diabetic retina, we discovered that IL-1β was predominant in retinal tissues of Ins2^Akita^-KO mice both at the transcript and protein levels. Although IL-1β expression in the retina has been previously documented (Grigsby et al., 2014; Scuderi et al., 2015), our data show a clear correlation between microglial activation, IL-1β production, and neuron damage in the diabetic retina of CX3CR1-KO mice. The fascinating biology of IL-1 (α and β), which as its name suggest was the first interleukin to be described, reveals many activities with participation in both innate and adaptive immune responses. IL-1 is expressed at low levels under normal conditions and requires induction at both transcriptional and translational levels. Elevated concentrations of IL-1 *in vitro* are toxic to neurons (Hailer et al., 2005; Ye et al., 2013), and elevation of IL-1 *in vivo* is associated with excessive growth of dystrophic neurites in Alzheimer’s disease models (Medel-Matus et al., 2014). Also, neurodegenerative effects of IL-1 are reported in brain injury (Murray et al., 2015), endotoxemia (Cardona et al., 2006b), and multiple sclerosis (Hooper-van et al., 2003; Heidary et al., 2014; Peelen et al., 2015). Similar to the findings presented in this study, in Alzheimer’s disease, IL-1 overexpression localized to areas of activated microglia associated with β-amyloid plaques (Griffin and Mrak, 2002) and also of relevance is the observation that IL-1β accelerates apoptosis of retinal capillary cells (Kowluru and Odenbach, 2004). IL-1β has also been implicated in promoting angiogenesis and blocking IL-1 improved endothelial dysfunction in streptozotocin-induced diabetic rats (Vallejo et al., 2014) and prevented choroidal neovascularization in laser-induced retinal degeneration models (Lavalette et al., 2011). However, we still need to clarify the effect of IL-1β blockade in neuronal loss and the processes related to improving vision loss.

In contrast to IL-1β that appears predominant in the diabetic KO groups, *Ins2^Akita^* mice with a CX3CR1-WT or KO genotype shared a set of five transcripts that were upregulated in both groups when compared with their nondiabetic counterpart. Of those, STAT3 showed a sixfold increase in Ins2^Akita^-WT and a fivefold increase in the Ins2^Akita^-KO group. STAT3 activation has been shown in the diabetic retina of streptozotocin treated rats (Al-Shabrawey et al., 2008). Moreover, the role of STAT3 in angiogenesis was reported by its actions in increasing VEGF expression in cardiac myocytes (Funamoto et al., 2000) and tumor settings (Wei et al., 2003; Xu et al., 2005). Our data highlight the potential relevance of STAT3 signal transduction pathways during DR due to its direct correlation with increased transcript and protein levels for VEGF at 10 weeks of age (Figure 4). The observed downregulation of hypoxia inducible factor 1 (HIF-1) on Ins2^Akita^-KO mice (Supplementary Table 7) also supports role of this inflammatory cascade in tissue damage during DR.

Our study also points to a potential critical communication pathway between microglia and astrocytes in the inflammatory process in the retina. Microglia secrete a broad spectrum of soluble mediators including cytokines, chemokines, and neurotrophic factors that act upon other cells, such as astrocytes, neurons, oligodendrocytes, and endothelial cells. It was reported that microglia regulate neuronal cell death in the retina, spinal cord, and brain during development (Bessis et al., 2007). Interactions between microglia and neurons that mediated suppression include CD200 receptor on microglia and CD200 expressed in neurons and oligodendrocytes (Walker et al., 2009). During CNS development, microglia play an active role engulfing synaptic material in a process termed synaptic pruning and referred as an innate surveillance function of resident microglia (Paolicelli et al., 2011). FKN/CX3CR1 has been involved in this process of synaptic pruning as evidenced by a reduced microglia density in developing CX3CR1-KO mice correlating with increase in the number of dendritic spines and immature synapsis (Paolicelli et al., 2011). However, little is known about how disruption of FKN/CX3CR1 signaling affects retinal microglia function. This issue is of particular relevance given the controversial observations linking a polymorphism in the CX3CR1 gene in humans with susceptibility to age-related macular degeneration (Tuo et al., 2004; Chan et al., 2005; Schaumberg et al., 2014). Biochemical studies suggested that the polymorphic variant CX3CR1^I249/M280^ exhibited adhesive defective properties, and therefore defective FKN signaling. Based on the observation of enhanced neuronal damage in CX3CR1-deficient mice, it would be of clinical relevance to dissect the effects of the human reference CX3CR1^V249/T280^ receptor and polymorphic variant ^I249/M280^ in microglial-mediated inflammation during DR.

Based on our data, comparison of microglia, neurons, IL-1β, and VEGF levels in diabetic WT and KO mice suggests that diabetes has pronounced and detrimental effects on retinal integrity and induces microglial reactivity and IL-1β release. Absence of CX3CR1 exaggerates these responses, but it is uncertain how this correlates directly to vision loss. We hypothesize that the more neuronal damage the more likelihood of visual impairment. The neuronal damage and inflammatory response appear then to be mediated in part by cytokines such as IL-1β, produced by microglia and astrocytes, and also to oxidative stress particularly in the absence of CX3CR1. Interestingly, in this diabetic model IL-1β and VEGF levels decreased at 20 weeks of age when compared with the 10-week time point. This suggests a potential regulatory mechanism. In agreement with this observation, vitreous VEGF levels in diabetic patients with proliferative diabetic retinopathy (PDR) appeared lower when compared with nonproliferative DR (NPDR) (Patel et al., 2006). This is paradoxical, since PDR is characterized by severe neovascularization, vitreous hemorrhage and tractional retinal detachment, and viewed as a more severe pathology (Cheung et al., 2010) due to the potential to cause total blindness. In fact, more than half of the PDR samples (Patel et al., 2006) displayed VEGF levels comparable to control nondiabetic samples and therefore highlights the challenges of anti-VEGF therapies.

The association of IL-1β release by astrocytes and microglia prompts the investigation of how exactly is hyperglycemia mediating such effects. Early release of IL-1β may be responsible for early vascular damage that is sustained by hyperactive microglia but as a diabetic state progresses, compensatory mechanisms with increase anti-inflammatory gene expression are then upregulated as means to control tissue damage. This is inferred by a predominant presence of anti-inflammatory genes (IL-13, TGF-β1, and BCL2) and signal transduction components (Supplementary Tables 8 and 9) at the 20-week time point. The transcript data also reveal potential regulatory pathways. For example, from our studies EphrinA1 (Epha1, Supplementary Table 7) was downregulated in Ins2^Akita^-KO tissues. This molecule was shown to inhibit VEGF in bovine retinal endothelial cells *in vitro* leading to decreased cell proliferation (Ojima et al., 2006). Moreover, *in vivo* injection of ephrinA1 suppressed ischemic retinal vascularization in a dose-dependent manner (Ojima et al., 2006). Therefore, multiple pathways can be explored to further understand the role of inflammation in the orchestrated response to balance neuroprotection and neurotoxicity in the diabetic retina. This study provides novel information on inflammatory processes elicited by hyperglycemia. Since vision loss is irreversible, targeting IL-1 and STAT3 and enhancing neuronal protection via FKN signaling among other pathways may provide beneficial alternatives for early stages of DR.

## Summary

Diabetic CX3CR1-KO retinas show pronounced IL-1β responses by astrocytes and microglia, correlating with increased numbers of activated microglial and decreased neuronal densities. CX3CR1 a key regulator of microglial function modulates inflammatory-mediated damage to neurons in the diabetic retina.
